# The Immediate Effect of Parachute-Resisted Gallop on Heart Rate, Running Speed and Stride Frequency in Dogs

**DOI:** 10.3390/ani11071983

**Published:** 2021-07-02

**Authors:** Sandra Hederstedt, Catherine McGowan, Ann Essner

**Affiliations:** 1 AniCura Falu Djursjukhus, Samueldalsvägen 2B, SE-791 61 Falun, Sweden; sandra.hederstedt@anicura.se; 2 School of Veterinary Science, The University of Liverpool, Leahurst Campus, Chester High Road, Neston, Wirral CH64 7TE, UK; cmcgowan@liverpool.ac.uk; 3 IVC Evidensia Djurkliniken Gefle, Norra Gatan 1, SE-803 21 Gävle, Sweden; 4 Department of Neuroscience, Uppsala University, Box 593, SE-751 24 Uppsala, Sweden

**Keywords:** dogs, heart rate, gallop, physical fitness, resistance exercise

## Abstract

**Simple Summary:**

Physical fitness is needed for canine athletes and working dogs to optimize their performance in various disciplines. Application of resistance on movements causes biomechanical and cardiorespiratory responses to physical exercise. However, there is still a lack of research on the effects of high-intensity resistance exercise on cardiorespiratory fitness components such as heart rate in canine athletes. In this article, we investigate the short-term effects of parachute-resisted galloping on heart rate, running speed and stride frequency. Healthy dogs of various breeds were extensively studied in five experimental single cases. The dogs ran on a straight 200 m course with and without resistive drag force applied by a parachute attached to their harness while heart rate, running speed and stride frequency were measured. Subsequently, the measurements were compared to baseline phases at rest. In the present trials we found that heart rate increases similarly with and without parachute-resistance while dogs galloped at lower speeds and with increased stride frequency with applied drag force. Our findings lead us to suggest that parachute-resisted galloping is a clinically applicable exercise in healthy dogs to achieve instant cardiorespiratory response.

**Abstract:**

Physical fitness is required for canine athletes and working dogs to optimize performance in various disciplines. There is a lack of research on the effects of resistance exercise on cardiorespiratory variables in dogs. The aim of this study was to investigate the immediate effects of parachute-resisted (PR) gallop on heart rate, running speed and stride frequency compared to unresisted (UR) gallop in dogs. Five N-of-1 trials RCTs with alternating interventions were implemented. Dogs ran on a 200 m course with and without resistive force applied by a parachute attached to their harness while cardiac inter-beat intervals (IBI), running speed and stride frequency were measured. The results were visually displayed and interpreted in graphs and percentage of non-overlapping data estimated effect size. Both interventions showed large effects on heart rate compared to resting values. Mean IBI increased (10–17%) during PR gallop compared to UR gallop although this change was small relative to decreased running speed (19–40%) and increased stride frequency (18–63%). Minimum IBI showed no difference between interventions indicating similar maximum heartbeat per minute. In conclusion, parachute-resistance resulted in dogs galloping at lower speeds at the same cardiorespiratory level of intensity, which may be useful in canine physical rehabilitation and fitness training.

## 1. Introduction

Physical fitness is a recognized requirement for canine sports athletes and working dogs to optimize performance in tasks that are presented to them in various disciplines [1,2]. Physical fitness refers to the physical capacity to carry out tasks “with vigor and alertness, without undue fatigue and with ample energy to enjoy leisure-time pursuits and to meet unforeseen emergencies” [3]. Canine physical fitness can be categorized into two groups: (1) health-related components of physical capacity (i.e., cardiorespiratory endurance, muscular endurance, muscular strength, body composition and mobility), and (2) skill-related components of physical capacity (i.e., agility, balance, coordination, speed, power, and reaction time) [1]. Each component of physical fitness needs to be properly addressed during physical exercise as they may have a beneficial impact the dogs’ longevity and ability to perform physical, athletic and olfactory tasks [1,4,5,6].

Repeated resisted exercise can be used to primarily improve muscular strength, speed, power and cardiorespiratory fitness [7,8]. The effects of drag force resisted interval training on movement technique, stride length and frequency, and velocity have been studied in humans. Parachute-resistance has been reported to be superior to other resistance methods as it has demonstrated the least change in running technique, decreased running speed by 4.4%, decreased risk of plateauing and improved sprint acceleration [8,9,10,11]. In galloping dogs, sagittal movements (i.e., flexion and extension) of the spine are important to help increase the stride length to achieve faster running speed at gallop. Since there is a lack of comparability between bipedal and quadrupedal locomotion the effects of parachute-resisted exercise on physical fitness components, including aerobic and anaerobic capacity, need to be further explored in canine athletes [12,13].

In fast gaits, such as in acceleration and suspension gallop, dogs need to generate whole-body muscle power [12,14,15] while oxygen consumption and heartbeats per minute increase. Normal adaptations occurring during physical exercise can be used as indicators of cardiorespiratory exercise intensity, as heartbeats per minute and oxygen uptake increase linearly with increased running speed in dogs [16,17,18]. There is a growing body of evidence on the response and effect of physical exercise in dogs. For example, resistance can be produced by using incline ground or exercise equipment to alter gravitational forces and increase muscle activity in dogs [19,20,21,22]. Application of external forces (e.g., water resistance) causes immediate biomechanical and cardiorespiratory responses to exercise [23,24,25]. High-intensity interval training with and without resistance may be possible alternatives to moderate-intensity continuous training in dogs with various functions (e.g., hunting, canicross, olfactory detection) and specific impairments (e.g., decreased muscle strength) to enhance physical fitness [5,26,27]. However, there is still hardly any research on high-intensity interval exercise and especially a lack of research on the immediate adaptations to drag force resisted exercise on cardiorespiratory fitness variables in canine athletes.

The aim of this study was to investigate the immediate effects of parachute-resisted gallop on heart rate (HR), running speed and stride frequency compared to unresisted gallop in dogs. Our hypothesis was that drag force executed by a parachute attached to a galloping dog would decrease inter-beat intervals, i.e., increase the amount of heart beats per minute, decrease running speed and increase stride frequency as compared to unresisted gallop.

## 2. Materials and Methods

### 2.1. Study Design

A series of five N-of-1 randomised controlled trials (RCT) (i.e., single case experimental design) with alternating interventions design; A_1_-B-A_2_-C-A_3_, was implemented [28,29,30,31]. Phase A included baseline measurements during which the dog rested in a familiar crate in a car. Phase B was gallop without resistance and phase C was gallop with drag force resistance applied by a parachute. The B and C phase were randomised by simple randomization (i.e., by tossing a coin) for each case. N-of-1 RCTs are defined as a variation of a randomised controlled trial focusing on the individual effects in the development of interventions, using detailed information obtained in many data points, which indicate individual effects prior to the implementation of larger studies on a group level [32,33,34].

### 2.2. Cases

All dogs enrolled in the studies were privately owned and prospectively recruited via social media in April 2018. The owners signed an informed owner consent, were informed about the study and possible withdrawal of participation at any time. The study was approved by the Local Ethical Committee in Uppsala (Dnr C17/2016). A total of 44 dog owners reported their interest. Inclusion criterion was body weight 20–40 kg and exclusion criteria were recorded or known diseases or injury in the last three months as well as ongoing medication administration. The owners graded their dog’s likelihood to return to the owner on a given signal, the dog’s ability to gallop 200 m and relax in new environment on an ordinal scale from zero to ten. Five dogs with high owner-reported grades were invited to take part of the study. Two of them declined and were exchanged with other participants.

### 2.3. Measurements and Equipment

**Figure 1:**
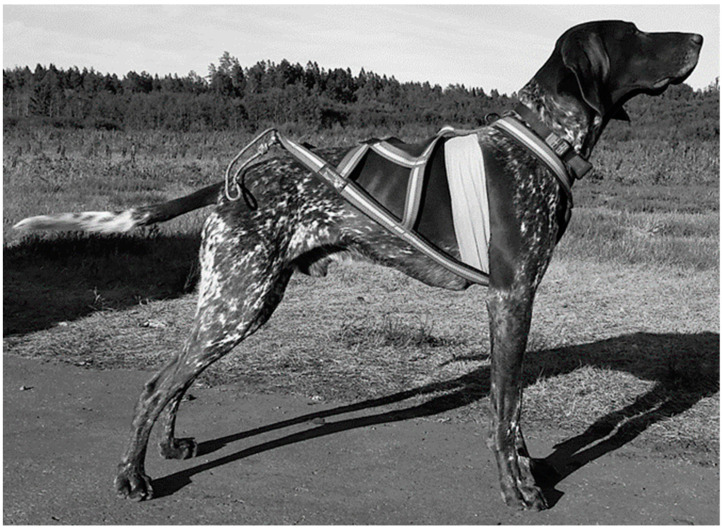
Illustrates a dog with nome-harness, heart rate sensor belt around the chest and heart rate monitor in a collar around the neck.

Heart rate measured as inter-beat intervals (IBI) (time in milliseconds between cardiac R-peaks) were recorded in all phases with Polar V800 HR monitor and H7 HR sensor [17,35,36]. Lower IBI corresponds to a higher frequency of heart beats per minute and vice versa. Dogs with long fur (case 1, 2 and 4) were clipped where the HR sensor was attached. The sensor was applied with a generous amount of electrode gel (Cefar-Compex Scandinavia AB, Malmö, Sweden) around the chest and secured with Vetflex (Jørgen Kruuse A/S, Langeskov, Denmark) [35]. The Polar V800 HR monitor was set in a collar around the dog’s neck (Figure 1). The dogs were fitted with a nome-harness of appropriate size prior data collection (Figure 1). Harness size was determined from the chest depth and body length when the harness was fully outstretched. The Power Chute (Power Systems PS, LLC, Knoxville, TN, USA) [11] 40″ × 40″ was attached at the end of the nome-harness to apply a resistive drag force while the dogs were galloping. Inter-beat interval recordings were collected at rest with the dogs in their car cages before the first run (A_1_) and following each phase B (A_2_) and phase C (A_3_). The IBI recordings at rest were started when the heart beats per minute did not vary by more than five for three subsequent minutes and were recorded for 30 s. Data collection took place in June 2018 outdoors on a straight 200 m pathway in an enclosed area at a local airport. A 200 m pathway was chosen since the dogs were to run at maximum self-selected speed and for enough time to generate many data points in the time series, while the owner was still in contact with their dog. Temperature, wind speed and humidity were registered from the airport weather station during all trials [37]. The running trials were video recorded with a GoPro Hero5 Black (60 frames per second) between 100 and 150 m.

### 2.4. Procedures

Subjects were asked to report for attendance on two separate occasions. The first session served to give information about the project and to familiarize the dogs and owners to the equipment. The dogs were examined by a veterinarian following a standard protocol and descriptive data, e.g., weight, body condition score [38] and muscle condition score [39] were collected. The owners were provided with a parachute and were instructed to practice in the home environment to acclimatize the dog before the trials.

The dogs were familiarized to the testing area, the test leaders and equipment. Each owner walked their dog on lead back and forth on the running course together with one of the authors as a warm-up and to optimize the dog’s focus during running.

The dog was put in its car cage with the boot cover open and the owner sitting beside the dog while baseline recordings were made. One of the authors walked the dog on lead 200 m away from the owner. The dog started from a standing position and the parachute was held off the ground for the resisted trials. Each owner called their dog on a given signal and the dog was released when the IBI recordings were started (Supplementary Materials Video S1; Dog galloping with and without parachute-resistance). Positive verbal reinforcement was allowed during running. All dogs had 15 min of rest between the intervention phases regardless of time needed to complete the baseline measurements.

### 2.5. Data Management and Analysis

Inter-beat interval data were transferred from the Polar V800 heart rate monitor to the Polar Flow software after each case recording. Polar Flow software was used to export IBIs as text files to the Windows based software Kubios HRV. Kubios HRV with a low threshold was used to indicate artefacts in the IBIs series [40]. Inter-beat interval data regarded as true artefacts were excluded from the analysis [36,41]. No data except artefacts were excluded in the baseline series. The first five seconds of phase B and phase C were excluded since five seconds were required to start the measurements and release the dog. The lengths of the IBI series in each intervention phase were based on estimated running time. Video recordings showing 50 m of the trials were used to calculate corresponding running times over 200 m, assuming constant running speed. The time length of the IBI series were corrected accordingly and IBI series from all five phases were smoothed using running medians of three [29]. Running speeds in kilometre per hour (km/h) and stride frequencies (i.e., number of strides) were calculated from the 50 m video recording. All statistical estimations and visual displays in graphs were performed in Microsoft Excel 365. Heart rate (i.e., IBI), IBI maximum, IBI minimum were reported as mean and standard deviation (SD). To increase readability, mean IBI was calculated and corresponded to heart beats per minute (BPM). 

For visual analysis IBIs (y-axis) from each dog were plotted against subsequent time during each trial (x-axis). Plotted data were displayed in graphs showing baseline and intervention phases in the randomised order. Data points were connected within time and phase, allowing evaluation of patterns within phases and changes between phases. The graphical displays of IBI data were visually inspected and interpreted based on trendline (slope, i.e., declination or inclination with and between phases), mean (changes from one phase to another), latency of change (i.e., how quickly change occurs) and shift in level (change in level from the last IBI in one phase to the next) [28,29,34,42]. Split-middle method was used to construct a linear plot in each phase [29].

In addition to the systematic visual analysis, the percentage of non-overlapping data (PND) was incorporated as a statistical measure of effect size of the differences between the alternating interventions [43,44,45]. The PND was obtained by counting the number of data points in phase B which do not overlap with the highest or lowest data points in phase C [43]. Percentage of non-overlapping data ranges from 0 to 100% with interpretation guidelines: >70% for effective intervention; 50–70% for questionable effectiveness and <50% for no observed effect [46].

## 3. Results

The series of N-of-1 trials involved five cases of different characteristics such as breed, sex, age and weight (Table 1). Two of the dogs were female, one of which was neutered, and three were neutered males. Age varied between 1.5 to 10 years old and body weight was 20–33 kg. All dogs had ideal Body Condition Score (4–5 out of 9) and normal Muscle Condition Score. Three of the dogs were randomised to parachute-resisted gallop as the first intervention and two dogs to unresisted gallop (Table 1). Two of the trials had to be repeated since the HR sensor fell off during one of the intervention phases in one dog and another dog stopped running for a couple of seconds when the wind filled the parachute but continued running after encouragement from the owner. Both dogs repeated their trials the next day without inconvenience.

The temperature ranged between 11.9–21.1 degrees Celsius and the wind speed was 4.4 m/s in all five dogs. All dogs were running towards the wind in order to fill the parachute. Humidity was between 31–41% and pressure 1013.5–1021.2 hPa [37]. The weather conditions during the trials are displayed in Table 1.

**Figure 2:**
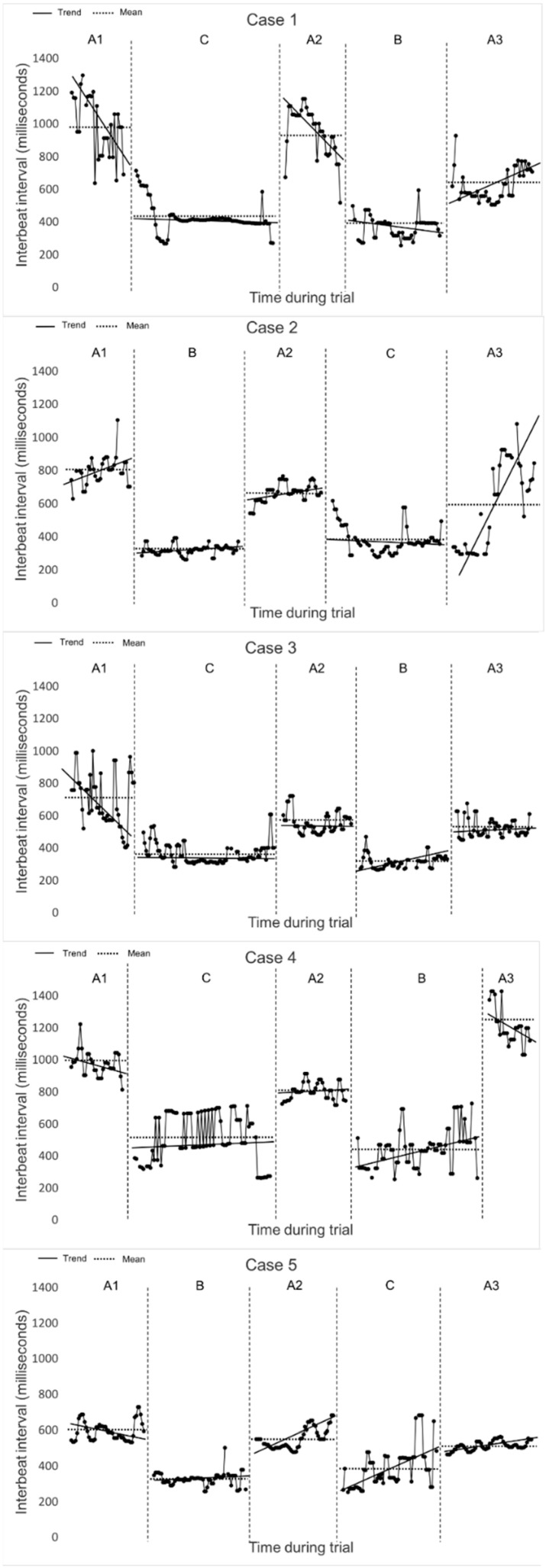
Graphs of the dogs’ series of inter-beat intervals during baselines A_1_, A_2_ and A_3_ and intervention phases B (i.e., gallop without parachute) and phase C (i.e., gallop with parachute). Y-axis shows inter-beat intervals in milliseconds and X-axis shows subsequent time during trial. Trendline (solid line) and mean inter-beat interval (dashed lines) are displayed.

The error rates in the IBI series were 0% to 16.7%. Artefacts were present in the intervention phases in four dogs and in various baseline phases in all dogs. All artefacts are displayed in the graphs as missing values (Figure 2).

The dogs were running 23–35 km/h in phase B and 14–27 km/h in phase C (Table 2), Hence, running speed decreased by 19–40% in phase C compared to phase B. The number of strides in the 50 m run were 13–21 in phase B and 17–31 in phase C. With parachute-resistance the stride frequency increased by 18–63%.

### 3.1. Visual Analysis of Graphic Displays

All cases showed a consistency in the overall pattern across phases. There was a rapid change in both B and C phases and the shift in level at point of phase change was downshifted from baseline to intervention phases and upshifted from intervention phases to baseline measurements in all cases. This confirms an immediate effect after the interventions was introduced corresponding to immediate cardiorespiratory effects in both interventions. The shift was greater from baseline to C phase compared to baseline to B phase regardless of the randomisation of the interventions in all cases except in case 2 in which it was the opposite. Trendlines showed very small to small change in slopes in both intervention phases in all cases. The trendline showed decelerating IBI (i.e., higher amount of heart beats per minute) in phase B in case 1 and accelerating IBI in case 2–5. Furthermore, there was a trend for decelerating IBI in phase C in case 1–3 and accelerating IBI in case 4 and 5. The trends were however very small and could in some intervention phases be seen as close to consistent. The trendlines during baseline had, in contrary, overall higher slopes. A_1_ had decelerating trend in all cases except case 2, which was accelerating. The trend in A_2_ was decelerating in case 1, accelerating in case 2 and 5 and close to consistent in case 3 and 4. A_3_ had a trend for accelerating IBI in all cases except in case 4 which had a pronounced decelerating slope. Overall, mean IBI was 10–17% increased (i.e., lesser amount of heart beats per minute) in phase C compared to phase B in all cases. Mean IBI was decreasing for each baseline during the trials; 4–25% decrease in mean IBI from A_1_ to A_2_ and 9–44% decrease in mean HR from A_2_ to A_3_ except in case 4 which had a 54% increase in mean IBI. IBI_min_ did not differ between interventions but IBI_max_ was increased in phase C in all cases by 11–111% compared to phase B.

When visually comparing non-overlapping data, we found that intervention phases were fully overlapping except for a few outlying data points in case 2 and 5, indicating similar effects in both interventions. A_3_ had the least non-overlapping data when comparing intervention phases to baseline phases, except in case 4 which had the greatest shift in A_3_ as previously mentioned (Table 2, Figure 2).

### 3.2. Effect Size

The PND between the intervention phases B and C ranged from 0 to 8.16%. The PND scores in case 1 to 5 were as follows: 8.16%; 5.36%; 0%; 6.06%; 1.64%. Altogether the PND scores indicate no observed effect on IBI between the interventions in any of the N-of-1 RCTs.

## 4. Discussion

The present study found that parachute-resisted galloping has acute effects on HR in dogs. The effects on HR were similar to unresisted gallop, but at lower running speed and increased stride frequency. The results showed higher mean IBI and IBI_max_ during parachute-resistance compared to unresisted gallop, the IBI_min_, however were similar between trials. The running speed decreased by 19–40% with parachute resistance, which is a greater difference than previously shown in humans [11]. This may be explained by longer acceleration time and shorter strides when running with a parachute, resulting in lower running speed and longer exercise time. As there is a linear relationship between speed of locomotion and heart beats per minute until maximum frequency, this series of trials indicates that parachute-resisted galloping results in a similar level of cardiorespiratory intensity but at lower running speed and higher stride frequency compared to unresisted gallop. This may be beneficial in exercising dogs as speed, frequency and exercise time are factors to take into account in exercise protocols and in order to prevent injuries.

It is discussed in the literature that the resistance produced from the parachute is highly variable depending on running speed and wind force [10,11]. Therefore, the dogs in this series of trials, allowed to run at a self-selected speed, were exposed to various doses of resistance. The dose–response relationship in parachute-resisted galloping needs to be further explored, especially considering the possible mediating and moderating effects of third variables (e.g., running speed and age of the dog).

The presented studies showed no difference between interventions regarding IBI_min_. The Polar HR monitor can only record IBI as low as 250 milliseconds (corresponding to maximum 239 BPM), which may limit the measurement of heart rate in dogs. There was however only one data point at 251 milliseconds in our series of N-of-1 RCTs, which strengthens the internal validity. A previous investigation had measured HR up to 237 BPM [17] with a Polar HR monitor device in dogs. The present series of N-of-1 RCTs reported contained 0% to 16.7% artefacts, which were subsequently excluded from the statistical estimations, but still displayed in the graphs. Canine inter-beat intervals measured with the Polar HR monitor has shown excellent validity if the IBI series contains less than 5% artefacts [36]. The presence of measurement errors may increase if the dogs have respiratory sinus arrhythmia during rest, hence during baselines in the dogs reported here [36]. Artefacts present during the galloping session may be explained by interrupted electrode contact due to extensive movements and muscle activity. The problem was not spread in this series of studies with large amounts of data points.

A limitation in these N-of-1 studies is that we had to re-estimate the running times since we were not able to stop the time measurements on Polar V800 instantly after the dogs finished their runs. To estimate the appropriate end of the IBI time series we had to use running time from the 50 m video recording. The video recordings were similar in all five studies and are therefore comparable.

Our research presents short-term effects of a novel intervention. The trials were completed without significant training of the subjects, indicating a feasible training method and potential clinical applicability. Parachute-resisted galloping applies to the overload principle which is fundamental in training programs for pet, athlete or working dogs. Short bouts of high intensity physical activity mirrors life and behaviour of dogs. High intensity interval exercise combined with parachute resistance as a novel intervention potentially targets cardiorespiratory capacity, muscle strength and power components of canine physical fitness.

In these single case experimental studies presented here we found that parachute-resisted galloping enables similar physiological stimulus and adaptation as compared to unrestricted high intensity galloping when it comes to cardiorespiratory capacity measured as heart rate. Notably the running speed decreased with parachute-resisted intervention in all five N-of-1 RCTs which may indicate that the dogs had to generate great propulsive forces. Drag force elicited by a parachute is related to speed and in these dogs the resistive force possibly decreases with slower self-selected running speed. Hence, the dogs might have adjusted the running speed due to the onset of muscle fatigue and inability of the muscles to generate force and speed of contraction. It was necessary to allow the owners to encourage their dogs throughout the trials. Regardless, the dogs were unable to maintain the same running speed with drag force resistance. This leaves us with several questions that require further investigation regarding long-term effects of parachute-resisted exercise, biomechanical changes, differences in time to full recovery and biomarkers of energy pathways. 

Finally, parachute-resisted running may be an alternative training method to uphill high intensity interval training. Although, there is a need for shorter diameter parachute equipment for dogs out of various sizes to increase usability of this training method.

## 5. Conclusions

In conclusion, interval exercise with parachute-resistance at gallop may be beneficial in canine fitness and rehabilitation training to produce slower running speed longer running times and increased stride frequency, at the same cardiorespiratory level of intensity as unresisted high intensity intervals.

## Figures and Tables

**Table 1:** Individual data and equipment randomisation for each N-of-1 randomised controlled trial. Weather conditions; wind, temperature, humidity and pressure during trials.

Case ID	1	2	3	4	5
Breed	Flatcoated Retriever	German Shepard	Australian Kelpie	Border Collie	German Shorthaired Pointer
Sex	female	Male neutered	male neutered	female neutered	male neutered
Age (years)	2	4	1.5	10	3.5
Weight (kg)	27.5	33.0	20.0	20.0	28.7
BCS (1–9)	4	4	5	4	4
MCS	normal	normal	normal	normal	normal
Equipment randomisation first run	parachute	without parachute	parachute	parachute	without parachute
Wind (m/s)	4.4	4.4	4.4	4.4	4.4
Temperature (Celsius)	21.1	11.9	13.2	21.1	21.1
Humidity (%)	41	38	31	41	41
Pressure (hPa)	1013.5	1021.2	1021.0	1014.0	1014.7

BCS = Body Condition Score, MCS = Muscle Condition Score, hPa = hectopascal.

**Table 2:** Results from inter-beat interval measurements (measured in milliseconds) in each baseline phase (A_1_, A_2_, A_3_), intervention phases B (i.e., gallop without parachute) and phase C (i.e., gallop with parachute), running times and stride frequency for each dog in the series of N-of-1 randomised controlled trials.

**Case 1**	**A_1_**	**C**	**A_2_**	**B**	**A_3_**
Mean IBI SD)	975 (355)	432 (314)	926 (110)	392 (128)	636 (62)
Mean BPM	62	139	65	153	94
IBI_min_	597	262	518	255	493
IBI_max_	1296	714	1195	641	932
Running speed (km/h)	NA	21	NA	35	NA
Stride frequency	NA	23	NA	16	NA
**Case 2**	**A_1_**	**B**	**A_2_**	**C**	**A_3_**
Mean IBI (SD)	807 (28)	327 (61)	667 (88)	385 (87)	596 (359)
Mean BPM	74	183	90	156	101
IBI_min_	605	254	539	261	289
IBI_max_	1164	454	788	696	1081
Running speed (km/h)	NA	38	NA	27	NA
Stride frequency	NA	13	NA	17	NA
**Case 3**	**A_1_**	**C**	**A_2_**	**B**	**A_3_**
Mean IBI (SD)	712 (32)	371 (66)	571 (37)	317 (42)	527 (12)
Mean BPM	84	162	105	189	114
IBI_min_	388	252	464	253	435
IBI_max_	1123	991	850	469	728
Running speed (km/h)	NA	19	NA	26	NA
Stride frequency	NA	25	NA	21	NA
**Case 4**	**A_1_**	**C**	**A_2_**	**B**	**A_3_**
Mean IBI (SD)	989 (100)	514 (81)	809 (14)	437 (177)	1242 (180)
Mean BPM	61	117	74	137	48
IBI_min_	813	258	708	251	1002
IBI_max_	1303	822	1000	726	1547
Running speed (km/h)	NA	14	NA	23	NA
Stride frequency	NA	31	NA	19	NA
**Case 5**	**A_1_**	**B**	**A_2_**	**C**	**A_3_**
Mean IBI (SD)	600 (37)	329 (57)	553 (93)	388 (153)	513 (59)
Mean BPM	100	182	108	155	117
IBI_min_	528	251	474	252	464
IBI_max_	837	501	737	682	567
Running speed (km/h)	NA	27	NA	22	NA
Stride frequency	NA	16	NA	20	NA

Mean inter-beat interval is presented as time between heart beats in milliseconds, SD = Standard deviations, BPM = Heartbeats per minute to corresponding mean inter-beat interval, IBI_min_ = Minimum inter-beat interval, IBI_max_ = Maximum inter-beat interval, km/h = kilometres per hour, Stride frequency= Number of strides in a 50 m run, NA = not applicable.

## Data Availability

The data are not publicly available due to ethical and privacy limitations based on the consent provided by the participants.

## Supplementary Materials

The following are available online at https://www.mdpi.com/article/10.3390/ani11071983/s1, Video S1: Dog galloping with and without parachute-resistance.
